# A dual-process model for cognitive training

**DOI:** 10.1038/s41539-023-00161-2

**Published:** 2023-05-06

**Authors:** Julia Ericson, Torkel Klingberg

**Affiliations:** Department of Neuroscience, Karolinska Institutet, Solna, Sweden

**Keywords:** Human behaviour, Cognitive control

## Abstract

A key goal in cognitive training research is understanding whether cognitive training enhances general cognitive capacity or provides only task-specific improvements. Here, we developed a quantitative model for describing the temporal dynamics of these two processes. We analyzed data from 1300 children enrolled in an 8 week working memory training program that included 5 transfer test sessions. Factor analyses suggested two separate processes: an early task-specific improvement, accounting for 44% of the total increase, and a slower capacity improvement. A hidden Markov model was then applied to individual training data, revealing that the task-specific improvement plateaued on the third day of training on average. Thus, training is not only task specific or transferable but a combination of the two. The models provide methods for quantifying and separating these processes, which is crucial for studying the effects of cognitive training and relating these effects to neural correlates.

## Introduction

For more than a century, a central question in cognitive psychology has concerned when, how and to what extent cognitive training transfers to nontrained tasks^1^. However, we still lack mathematical models to describe the processes occurring during cognitive training^2,3^.

In an attempt to clarify this issue, cognitive transfer has been categorized as either “near” or “far”^2,4^. Near transfer refers to improvement on the trained task or tasks very similar to it. Such improvement could be the result of implicit processes, including perceptual improvements with training^5^ or automatization of stimulus-response rules^6^. Improvements on the trained task could also be due to explicit strategies, such as associating numbers in working memory (WM) with long-term memories, which leads to large, rapid improvements in the trained task but does not transfer to other tasks^7^. Far transfer, on the other hand, refers to improvement of a more general capacity. Capacity does not necessarily mean general intelligence (g) but could refer to a broader ability such as flexibility, attention, inhibitory ability, visuospatial WM capacity, or spatial ability. Such improvements, although not synonymous with g, are still useful for a wider range of nontrained tasks^2,3,8^. However, a weakness of the near/far categorization is that this division is often imprecise or arbitrary. Confirmatory factor analysis (CFA) is one possible method for characterizing and quantifying transfer^9–12^.

Studying the temporal dynamics of cognitive training is another approach to separate different underlying processes. For example, in motor skill training, the study of temporal dynamics has led to the identification of two separate learning processes that occur at different timescales^13^. First, fast improvement, which is related to increases in striatal activity, has been linked to the development of task-specific routines. Second, a slower reorganization of the motor cortex emerges after weeks of training^14^. Similar results, with a distinction between striatal and cortical involvement, have been reported in associative learning studies^15^.

Temporal dynamics have also been utilized to identify different phases of mathematical skill acquisition. Tenison and Anderson^16^ fitted a piecewise continuous power function to mathematical learning performance data using a hidden Markov model (HMM). At each time point, the HMM evaluates the probability of transitioning from one learning phase to the next, where each phase has a distinct intercept and learning rate. Tenison and Anderson found 3 mathematical skill acquisition phases. Importantly, the 3 phases were identified only when the HMM was fitted to individual training data, while only one phase was observed at the group level. This finding highlights the importance of analyzing temporal dynamics at the individual level, as averaging may provide a false impression of smoothness^17–19^.

Temporal dynamics have thus been used to identify different learning phases, reflecting distinct neural processes, in motor, associative and mathematical learning, but it has not yet been applied to cognitive training. In this study, we hypothesized that cognitive training involves at least two different processes: a task specific improvement and a capacity improvement which generalizes to other cognitive tasks. We aimed to identify these processes in two ways: (1) factor analysis of training performance combined with repeated tests of transfer tasks; (2) HMM analysis of the finer temporal dynamics of individual training curves.

**Fig. 1:**
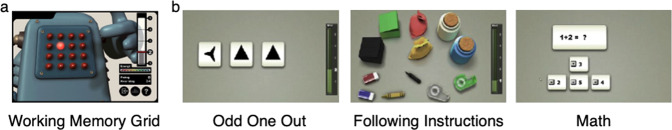
The training and transfer tasks. **a** The trained task (Grid). A sequence of red light bulbs light up on the robot and the task is to remember the order of the sequence and then reproduce it. **b** The 3 transfer tasks (Odd One Out, Following Instructions and Math). In Odd One Out, the child is shown a sequence of cues. Each cue includes 3 shapes of which one is odd, as shown in the figure. The task is to remember the position of the odd shape for each cue in the sequence. In Following Instructions, a sequence of verbal instructions are given asking the child to move specific items on the screen (e.g., “Click on the green eraser, then drag the black crayon to the yellow box”, which would be a trial on level 3). In Math, arithmetic problems with 4 answer choices are displayed on the screen. The child answers by pressing the arrow-key on the keyboard corresponding to the position of the correct answer choice on the screen.

We included data from 1300 children, ages 9 to 11, who participated in 40 days of WM training. The training tasks were mainly spatial WM tasks such as Grid^20^ (Fig. 1a). They also completed 5 testing sessions (T1–T5) of 3 transfer tasks (Fig. 1b). The first task was the odd-one-out^21^ (OOO), which did not share any perceptual similarities with the trained visuo-spatial tasks, but still relied on visuo-spatial WM capacity. The second task was following instructions^22^ (FI). In this task, children were given a verbal instruction of how to move objects on the screen. We hypothesized that spatial WM representations are important also for verbal instructions^23,24^. The third task, Math, was an arithmetic task of addition under time pressure. Since spatial representations are important for mathematics^25–28^, and WM training has previously been shown to improve mathematical performance and learning^12,29^, we expected an improvement here too.

## Results

### Confirmatory factor analysis

**Fig. 2:**
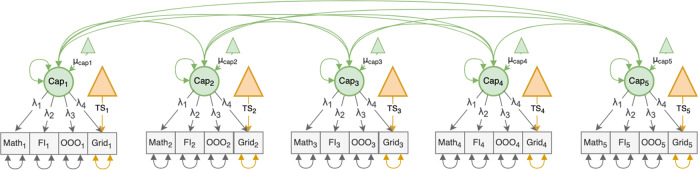
CFA model with one latent capacity factor (Cap) and one task-specific intercept (TS) for Grid. The model includes the 3 transfer tasks (Math, FI, and OOO) as well as the trained task (Grid). Note that the model also includes correlations between the same task at different time points; however, these correlations were excluded from the figure to reduce clutter.

First, we used CFA to examine whether the improvement on Grid could be separated into one task-specific (TS) process and a more general capacity (Cap) process which transfers to other types of cognitive tasks (Fig. 2). Performance data from Grid and the 3 transfer tasks were entered into a factor model, which was tested for strict longitudinal measurement invariance; that is, the factor loadings, task intercepts and residual variances should remain constant through all measurement sessions.

Strict measurement invariance is necessary for a meaningful comparison of latent factors at different points in time^30^. Nevertheless, some intercepts and residual variances can be allowed to change if there is a valid reason. Here, we did not expect task-specific improvements in the transfer tasks, as these tasks were only performed on a few occasions, while we did expect task specific changes in the trained task. Thus, Grid was exempted from the invariance criteria.

As hypothesized, the model showed strict measurement invariance when the intercept and residual variances for Grid were excluded from the constraint, while the test failed if they were included. The root-mean-square error of approximation (RMSEA) was 0.036, and the comparative fit index (CFI) was 0.984 for the model with free intercept and residual variances for Grid. Even though Grid did not show strict measurement invariance throughout the whole training period, we investigated if either strict or strong measurement invariance would hold during parts of the period. Strong measurement invariance implies that only the intercepts need to be constant, but the residual variances can vary. This analysis showed that Grid could be included in the constraint for strong measurement invariance from T2 to T5 without significantly reducing the fit (RMSEA = 0.038, CFI = 0.982). As a comparison, strong measurement invariance did not hold when the constraint was placed only on T1 to T2 (RMSEA = 0.051, CFI = 0.969). Strict measurement invariance did not hold for any period.

**Fig. 3:**
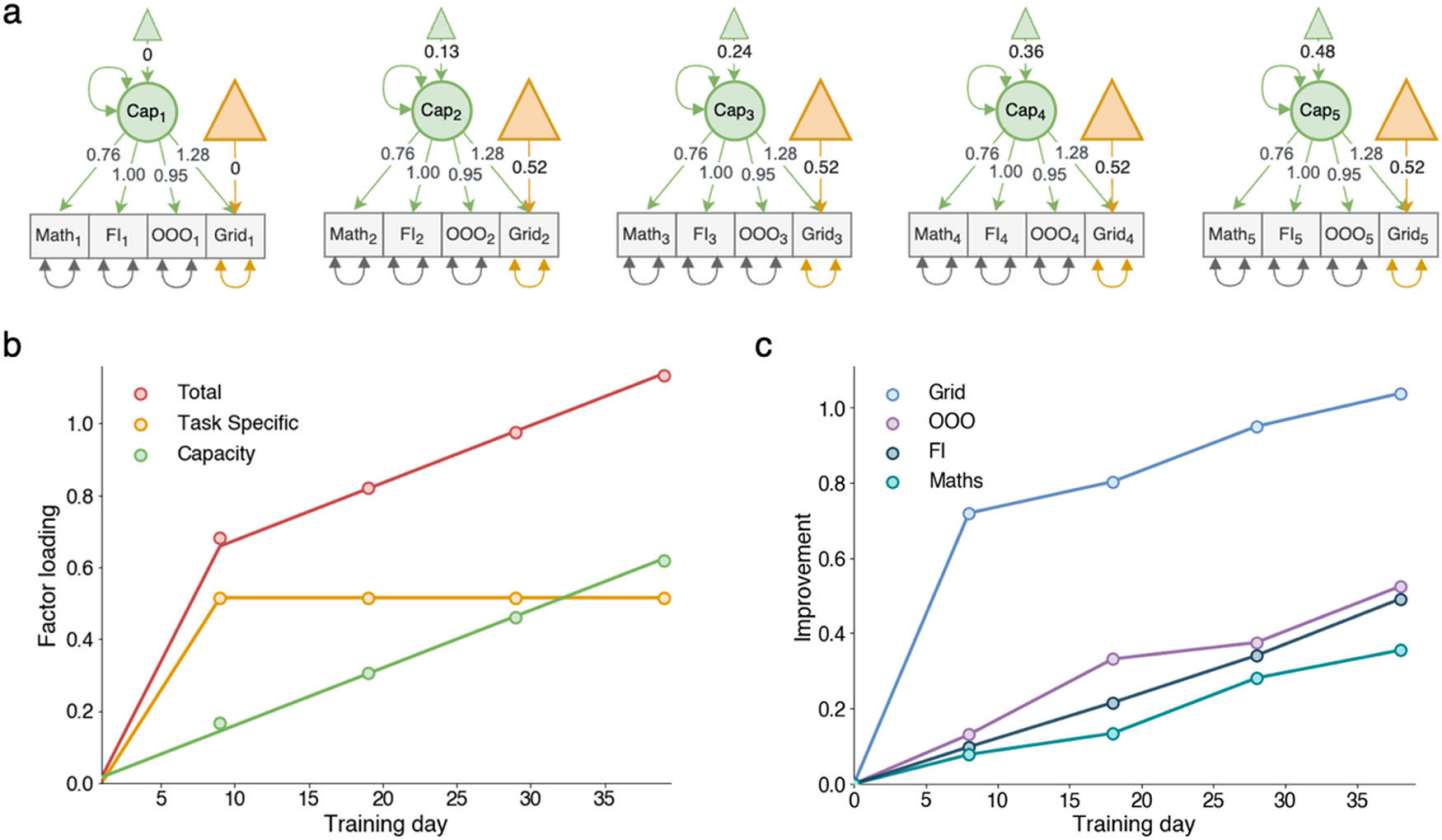
Results from the CFA. **a** Final loadings from the CFA model. The task-specific intercepts of the transfer tasks were all zero. **b** The change in the latent capacity and task specific intercept for Grid as well as the total combined improvement on Grid (1.28*Cap + TS). **c** The average improvement on Grid and the transfer tasks, standardized with the respect to T1 performance for each task ((Performance_Ti_ - M_T1_)/SD_T1_).

The final model (Fig. 3a)—with strict measurement invariance for the transfer tasks, strong measurement invariance for Grid between T2 and T5, and weak measurement invariance for Grid between T1 and T2—suggested two things. First, the transfer improvements were driven by Cap alone while the improvement on Grid was a combination of both Cap and TS. Second, Cap increased continuously throughout the training period while TS increased only between T1 and T2 (Fig. 3b). This result is also consistent with the behavioral data (Fig. 3c).

Using the final model, we estimated individual Cap and TS values for each subject. By fitting a line through Cap, we measured the rate of capacity improvement for each subject. The average R-squared value for the regression of Cap was 0.90. Moreover, we defined the task-specific improvement for each subject as the TS difference between T2 and T1.

### Multivariate latent growth curves

**Fig. 4:**
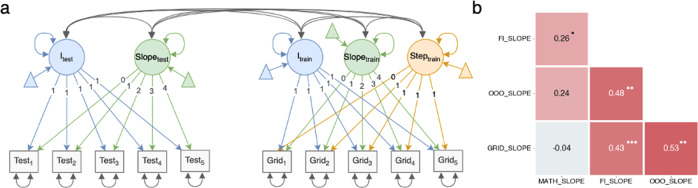
Results from the multivariate latent growth curve model. **a** The multivariate latent growth curve model. The figure shows only one transfer task. However, all transfer tasks were included in the model. **b** Heatmap of correlations between the slopes in the model. *p ≤ 0.05, **p ≤ 0.01, ***p ≤ 0.001.

The CFA model showed that the average capacity increase was linear, but to show that this also applies to individuals, the capacity improvement on Grid should correlate with the improvements on the transfer tasks as well^31^. To investigate these correlations, we modeled the improvement on the 4 tasks as latent growth curves, with one curve representing each task. The latent growth curves of the transfer tasks were modeled as linear functions with an intercept and a slope. For Grid, the previous CFA suggested that the improvement needed two components—a linear function and a step function to capture the increase between T1 and T2. Thus, the latent growth curve of Grid also included a step function in addition to the intercept and slope (Fig. 4a).

All correlations between the latent factors were estimated in one model. The data fitted the model well (RMSEA = 0.034, CFI = 0.987). Moreover, we observed significant correlations between the slopes of the trained task and two of the transfer tasks (Fig. 4b), OOO (0.53 ± 0.17) and FI (0.43 ± 0.13), validating our hypothesis that the slope of Grid was related to the slopes of OOO and FI. Conversely, no significant correlation between the slope of Grid and the slope of Math (−0.04 ± 0.10) was observed; thus, we cannot determine if the improvement in Math was actually driven by the improvement in the Grid task using this method.

### Fitting individual learning curves to daily training data

The CFA model, which was based on sparsely collected transfer measures, suggested that training improvement included two different processes: task-specific improvement and capacity improvement. Capacity improvement was present throughout the training period, while task-specific improvement occurred between T1 and T2. Next, we assumed that these processes would also be reflected in the individual learning curves of the trained task Grid. We therefore developed a mathematical model, fitted to the daily performance data on Grid, which could: (1) reflect this dual-process improvement, (2) correlate with the CFA measures, and (3) provide more detailed information about the temporal dynamics of these processes.

The CFA model suggested not only a two-factor model but also two different phases. The first phase included both task-specific improvement and capacity improvement, while the second phase included only capacity improvement. Mathematically, the CFA results can be described by a piecewise linear function with the following two phases:

1 $${Phase}\,1:y={\beta }_{{cap}}t+{\beta }_{{TS}}t+\alpha$$

2 $${Phase}\,2:y={\beta }_{{cap}}t+{\varDelta }_{{TS}}+\alpha$$

where ${\beta }_{{cap}}$ is the capacity improvement rate, ${\beta }_{{TS}}$ is the task-specific improvement rate, and $y$ is the performance. ${\varDelta }_{{TS}}$ is the total task-specific improvement at the point where the model transitions from phase 1 to phase 2 and $\alpha$ is the initial performance level before training.

**Fig. 5:**
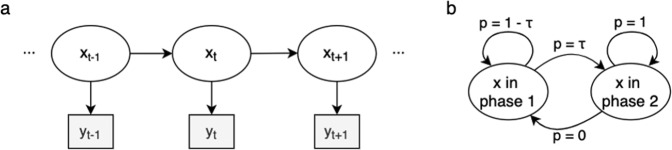
Hidden Markov model. **a** The dependencies of the observed variables **y** and hidden states **x**. **b** The transition probabilities between the hidden states **x**.

To fit the piecewise linear model, we used an HMM^16^. An HMM is a probabilistic graphical model where the state of the system is hidden but a variable related to the hidden state can be measured. In this case, the observed measurement is the performance *y* on each day, and the hidden state is the current phase. The HMM assumes that the variable observed at time *t* depends only on the hidden state at *t*, and that the hidden state at *t* depends only on the hidden state at *t*–1 (Fig. 5a).

**Fig. 6:**
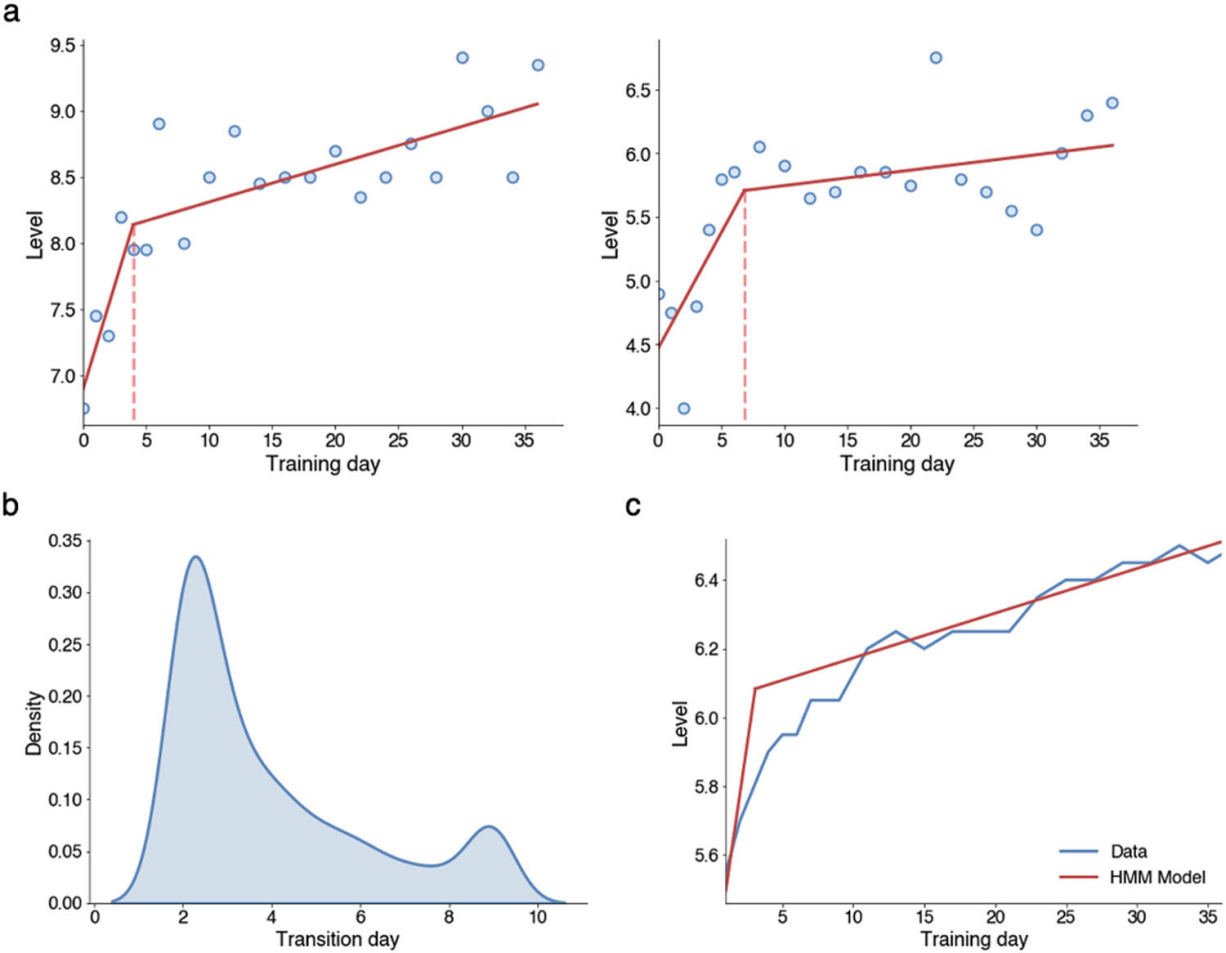
Results from the hidden Markov model. **a** Example of the piecewise linear model fitted to two different subjects. The dotted line marks the transition between phase 1 and phase 2. **b** A density plot of the estimated transition days for the population. **c** The averaged training data for the whole population and piecewise linear model with average parameter values.

To estimate the most likely state at each time point, 3 probabilities are needed. First, we need to determine the probability of an observation given a hidden state. This probability, which is calculated based on Eqs. (1) and (2), is included in Method. Second, the probability of transitioning between phases must be obtained. We call the probability of transitioning from phase 1 to phase 2 τ, meaning that the probability of remaining in phase 1 is 1–τ. Furthermore, the probability of returning to phase 1 from phase 2 is 0 (Fig. 5b). Third, we need to know the probabilities for starting in either of the hidden states. Here, we assumed that phase 1 is always the starting phase. The probability parameters were optimized using expectation maximization^32^. We fitted one learning curve for each of the 1300 subjects. Figure 6a shows examples of two learning curves.

Next, we correlated the parameters ${\beta }_{{cap}}$ and ${\varDelta }_{{TS}}$ with the two individual parameters extracted from the CFA model. We found that ${\varDelta }_{{TS}}$ in the HMM was significantly correlated with TS in the CFA model (*r* = 0.78, *p* < 10^−5^, Table 1) and slightly correlated with Cap (*r* = 0.21, *p* < 10^−5^). ${\beta }_{{cap}}$ in the HMM was correlated only with Cap in the model CFA (*r* = 0.45, *p* < 10^−5^) and not with TS (r = -0.01, *p* = 0.72). Thus, the improvement rates based on the daily training data were consistent with the CFA estimates. Moreover, the median day for transitioning was day 3.05, with quartiles of 2.13 and 5.18 (Fig. 6b). The transition day density plot in Fig. 6b has a second peak at training day 9 (day 10 in total). This is due to the constraint placed on the transition day (see Method) and does not imply that the distribution is bimodal. Finally, Fig. 6c compares the average performance and the average model fit (${{\beta }_{{TS}}=0.269,\beta }_{{cap}}=0.013,{\alpha }=5.497,\tau =0.481$).

**Table 1:** Pearson correlation between individual capacity and task-specific improvement extracted from the CFA model and HMM, ***p ≤ 0.001.

CFA	HMM	
	Task-specific	Capacity
Task-specific	0.78***	−0.01
Capacity	0.21***	0.45***

### Simulations

To test the reliability of the model, we fitted it to simulated data where the true parameters for each subject were known. We investigated whether the estimations were biased and assessed the size of the prediction errors. A total of 1000 simulated subjects with parameter values sampled from uniform distributions were used. Gaussian noise was added to the performance $y$, and the noise level on each day was approximated according to the average estimated noise level in the real data on that day. The biases for the 3 parameters were −0.00039 for ${\beta }_{{cap}}$, 0.092 for ${\beta }_{{TS}}$ and −0.14 for $\alpha$. The respective root-mean-square errors (RMSE) were 0.0069 for ${\beta }_{{cap}}$, 0.22 for ${\beta }_{{TS}}$ and 0.39 for $\alpha$. The bias in the transition day was −0.37 days, with an RMSE of 0.58. In 88% of the cases, the model predicted the transition day within one day of the true transition day. In 94% of cases the transitions were predicted within two days of the true transition day.

## Discussion

Here we aimed to identify and quantify the different processes that occur during WM training and to characterize the time dynamics of these processes. First, a factor analysis confirmed our hypothesis that there are two different processes occurring simultaneously during training: (1) an early task specific improvement reaching a plateau, and (2) a slow, linear improvement of a capacity which generalized not only to a non-trained spatial WM task, but also to a verbal WM task and a test of mathematics.

Secondly, we used an HMM to describe the temporal dynamics of individual training performance more precisely. This analysis indicated that the task-specific improvement in average occurred during the first 3 days, after which it plateaued. However, there was an inter-individual variability in the timing of when the task-specific improvement reached the ceiling. Simulation studies showed that the HMM was able to identify this point within a day in 88% of cases. In contrast to task-specific improvement, the capacity improvement increased linearly throughout the 8 weeks of training. The task-specific and capacity improvements estimated by the HMM were significantly correlated with the estimates obtained from the CFA model.

Importantly, the task-specific improvement was identified only when analyzing individual learning curves and was not apparent in the average performance data, which had a considerably smoother appearance (Fig. 6c). This result is consistent with previous research, which has highlighted that averaged data can create smoothing effects that do not accurately reflect individual trajectories^16–18^.

The capacity factor loaded both on the trained task and on the 3 transfer tasks: OOO, FI, and Math. Improvements on these transfer tasks are consistent with findings in prior studies that used the same WM training method and compared the outcome to active^33,34^ or passive control groups^35^. In the study by Bergman-Nutley and Klingberg^35^, the effect size was 0.69 for FI, 0.60 for OOO and 0.44 for Math, which are slightly larger than our effect sizes of 0.48 for FI, 0.46 for OOO and 0.37 for Math.

So, what mental ability could the capacity factor correspond to? Grid and OOO are both visuospatial tasks, but visuospatial representations are also important for cognition when stimuli are presented verbally^23,24^, as in our FI task. For example, the subjects could translate the verbal instructions to a spatial representation of planned movements. Similarly, visuospatial WM representations are an integral part of mathematical reasoning^26^, and training on visuo-spatial WM training improves mathematical performance in the average population^12,29^ (but see also^36^). We therefore speculate that the capacity factor we identified corresponds to a general visuospatial WM capacity, which is important for many cognitive tasks.

Two processes that operate at different time scales have already been proposed as a model for motor learning^13^. Early striatal activation has been linked to rapid development in task-specific routines, which was then followed by slower cortical changes^14^. A similar mechanism could apply to cognitive training. Here, imaging studies have identified activation in both the striatum and frontoparietal cortex^37–40^. For example, Kühn et al.^39^ observed a rapid increase in activity in the left striatum during n-back training that was present in both the training and active control groups. Striatal volume has also been shown to predict early improvement during video game skill acquisition^41^. This suggests that striatal activity increases could be related to task exposure and automatization of stimulus-response rules, generating the task-specific increase observed in our study.

Frontoparietal activity is correlated with WM capacity^40,42–44^ and cortical plasticity in this network could underly a capacity improvement. In general, cortical plasticity is known to be a slow process operating on a time scale of weeks or months^45^. Moreover, many cognitive training studies in both humans and macaques have found slow neural changes that are present throughout the whole training period, even when most of the behavioral improvement occurs in the beginning of the training period^37,46,47^. The two processes that we identified could thus have different neural correlates and correspond to an early striatal phase related to the task-specific improvement, and a slower cortical phase underlying the change in capacity.

There are several limitations of the current study. Only 3 transfer tasks were used, and it is possible that additional transfer tasks would have revealed more than one latent factor. Likewise, a longer training period could potentially yield more than two phases^48^. Furthermore, it is unclear to us why there was not a significant correlation between the latent slopes of Math and Grid in our latent growth models. A non-linear model for individual Math improvements, a model capturing delayed improvement, or a larger dataset^49^, might be necessary.

The relatively high number of repetitions may raise concerns regarding test-retest effects in the transfer tasks. However, we do not believe this is the case for several reasons. First, the subjects took the transfer tests twice before training, and the first attempt was discarded from the analysis, to minimize test-retest effects. Second, the strict longitudinal measurement invariance in our model should ensure that no test-retest effects occur during training^50^. Third, we compared the capacity increase in our CFA model to the increase in the controlled study by Bergman-Nutley and Klingberg^35^ and found that the CFA model gave a more conservative estimate.

A limitation with CFA is that the models depend on various assumptions. In our case, a model with completely free intercepts for the trained task would have suggested that the task specific improvement peaked at T2, followed by a small decrease rather than a plateau. This would not have changed the conclusions significantly: the capacity factor of the CFA would still increase linearly, the transition day would still be day 3, and the capacity factor of the CFA would still be significantly correlated with the capacity slope of the HMM. However, it seems implausible that the subjects would get worse with practice after a few days. We therefore do not think that this decrease is an actual reduction in skill, but perhaps a decreased motivation for the trained task, or random noise. Thus, we chose the most conceptually plausible model, with a fixed intercept from T2.

In summary, the HMM and the CFA model both identified a fast, task-specific improvement and a transferable capacity improvement, which was slower but constant throughout the training period. Altogether, these time courses resembled logarithmically shaped training curves. In the future, we believe that the proposed models may be useful for elucidating neural processes associated with task-specific and capacity changes.

## Method

### Participants

We used data from 1667 children, age 9 to 11 (577 9-year-olds, 631 10-year-olds and 459 11-year-olds), collected on Cogmed between 2016 and 2019. The data were anonymous, where data for each child were marked with an index which could not be connected with the identity of the child. No data other than age and task performance was recorded. Ethical approval for the study design was obtained from the Swedish Ethics Review Agency. The Swedish Ethics Review Agency explicitly waived the requirement for informed consent as all data was anonymous and did not contain any personal or sensitive information, and the children were not exposed of any possible harm. Furthermore, the children were not recruited for the research study specifically but underwent the Cogmed training program for their own benefit.

### Behavioral data from cognitive training

The cognitive training software used here was Cogmed (https://www.cogmed.com), which includes 12 closely related WM tasks practiced over 8 weeks. The training sessions were 25 min long and scheduled for every weekday, except for 6 days, which were set aside for transfer testing. No training was done on weekends. The training was adaptive, such that the children always worked at a level close to their capacity limit. To increase variability, 8 of the 12 tasks were practiced in each session. Grid, which was included in 22 sessions, was practiced the most and therefore, it was used as the trained task for the subsequent analyses.

Transfer testing was performed on days 1, 2, 10, 20, 30, and 40. Data from day 1 was discarded as this was a preparatory session allowing the children to familiarize themselves with the tasks, which was necessary for the non-supervised testing format. The transfer test on day 1 was then omitted, and day 2 was referred to as Testing Day 1 (T1).

The daily performance for each training task was defined as the highest level reached during a session. For Grid, the level corresponded to the number of items in the presented sequence. For the transfer tasks OOO and FI, the level was also defined as the sequence length, where the level increased until the child had answered two questions incorrectly at the same level. The previous level became the final score for the test session. During the math test, the participants had one minute to answer as many problems as possible, and the number of correctly answered questions was used as the final score.

Of the 1667 children, 366 children had missed 6 or more tests, which corresponded to two full test sessions. These were excluded from the analysis. Other missing data points were imputed using the *k*-nearest neighbor approach, where the mean value of the 35 nearest neighbors was used to impute the missing value.

### Factor analysis

For the factor analysis, the data of each task were standardized with respect to the mean and standard deviation of the performance on that task at the first time point (T1). The analysis was implemented in R using the package Lavaan^51^ and models were fitted using the robust maximum likelihood estimator (MLM). The cut-off values for the fit of all models was set to CFI = 0.95 and RMSEA = 0.06^52^.

### CFA

We employed CFA to examine the temporal evolution of the latent factor and the intercept of the trained task. The latent factor was constructed from 3 transfer tasks and the trained task. To establish a scale for the model, we fixed the sum of the 4 factor loadings to 4 and the sum of the 3 transfer task intercepts to 0 at each time point. However, we did not include the intercept of the trained task in this constraint as we were interested in examining changes specific to that task.

We then tested the model for longitudinal measurement invariance, which implies 3 successively stricter constraints placed on the model. First, weak measurement invariance tests if the factor loadings can be fixed across time. Second, strong measurement invariance tests if the intercepts can be fixed. Finally, strict measurement invariance tests if the residual variances can be fixed. Here, we also constrained the task covariances such that cov(T1, T2) = cov(T2, T3) = cov(T3, T4), cov(T1, T3) = cov(T2, T4) = cov(T3, T5), and cov(T1, T4) = cov(T2, T5). For invariance, the increase in the RMSEA was required to be less than 0.015, and the decrease in the CFI less than 0.010 in each step^53^.

In the weak measurement invariance test, all four tasks had constrained factor loadings. In subsequent steps, we assumed that task-specific effects could impact the intercepts and residual variances of the trained task, so these parameters were initially not constrained. We then examined whether strict or strong measurement invariance would also apply to the trained task, both over the entire training period and within subparts.

From the final model, we extracted the predicted factor values for each participant to calculate the individual improvements as estimated by the model.

### Latent growth models

We utilized a latent growth model to examine the correlation of improvements on an individual level (Fig. 4a). The factor loadings from the linear functions and the step function were fixed while all other variables were estimated, including task intercepts, task variances and covariances, and factor variances and covariances. To ensure that the model converged, we assumed that the Grid residual variance was constant. This was necessary as the model was unable to differentiate the residual variance of the Grid task at T1 from the variances of the Grid intercept and step factor.

### Piecewise linear model

We fitted the piecewise linear learning curve using an HMM. Here, we assumed that the learning trajectory of Grid could be described using the following function with two consecutive phases:

3 $${Phase}\,1:y={\beta }_{{cap}}t+{\beta }_{{TS}}t+\alpha +{\rm{w}}$$

4 $${Phase}\,2:y={\beta }_{{cap}}t+{\varDelta }_{{TS}}+\alpha +{\rm{w}}$$

where *w* is white noise with a standard deviation of σ. The probability of observing a certain performance *y* on day *t* is

5 $$p\left({x}_{t}\right)=N({\beta }_{{cap}}t+{\beta }_{{TS}}t+\alpha ,\sigma )$$

if *t* is in phase 1 and

6 $$p\left({x}_{t}\right)=N({\beta }_{{cap}}t+{\varDelta }_{{TS}}+\alpha ,\sigma )$$

if *t* is in phase 2. Here, *x*_*t*_ is the phase on the given day. The probability of transitioning from phase 1 to phase 2 is calculated as

7 $$p({x}_{t}|{x}_{t-1})=\tau .$$

Therefore, ${\varDelta }_{{TS}}=\frac{{\beta }_{{TS}}}{\tau }$. The probability of the transition occurring on day *t* can be expressed as

8 $$p\left(t{rans}=t\right)={(1-\tau )}^{t}\cdot p\left(x=1\right)\cdot \tau \cdot p\left(x=2\right).$$

Hence, the probability of being in phase 1 at time *t* is

9 $$p\left({x}_{t}=1|{y}_{1},\ldots ,{y}_{N}\right)={{\Sigma }}_{n=t}^{N}p({trans}=n)$$

while the probability of being in phase 2 is

10 $$p({x}_{t}=2|\,{y}_{1},\ldots ,{y}_{N})={\Sigma }_{n=1}^{t-1}p\left({trans}=n\right).$$

These phase probabilities for each time point are then used in the expectation maximization algorithm^32^ to optimize the model parameters in an iterative process. Each iteration includes two steps. First, the phase probabilities

11 $$p({x|y},{\theta }_{k})$$

are calculated, where ${\theta }_{k}$ represents the model parameters at iteration *k*. Next, the parameters $\theta$ are optimized to maximize the expected value of the observed sequence ***y*** and phase ***x*** with respect to $p({x|y},{\theta }_{k})$:

12 $${\theta }_{k+1}={\arg }{E}_{p(x{\rm{|}}y,{\theta }_{k})}[{logp}(x,y{\rm{|}}{\theta }_{k})]$$

The iterations continue until the model converges. The maximization step was performed using the function minimize in the Python package SciPy.

The transition day was constrained by the results of the CFA model such that the latest allowed transition day was day 10. This impacted 4.5% of the population which had a transition day above 10 days without the constraint in place. After parameter fitting, one participant was also removed since their ${\beta }_{{cap}}$ value was approximately 10 times larger than the standard deviation of the population.

### Simulations

To validate our optimization algorithm, we simulated 1000 subjects using the piecewise linear model, and the parameters were sampled from a uniform distribution between 2.0 and 4.0 for $\alpha$, 0.8–1.6 for ${\beta }_{{TS}}$ and 0.01–0.1 for ${\beta }_{{cap}}$. The transition day was sampled to occur between days one and ten. Zero mean Gaussian noise was added to the final performance *y* for each subject and day. The noise level varied from day to day to reflect the noise level in the data set on the given day. Thereafter, we derived the original parameter values of each subject using the HMM.

### Reporting summary

Further information on research design is available in the Nature Research Reporting Summary linked to this article.

## Supplementary information

Reporting Summary

## Data Availability

The data is available upon reasonable request to corresponding authors.

## Code availability

The code can be accessed at https://github.com/juliaericson/Working-Memory-Training-Learning-Curves.

## References

[1] Woodworth RS, Thorndike E (1901). The influence of improvement in one mental function upon the efficiency of other functions. (I). Psychol. Rev..

[2] Katz B, Shah P, Meyer DE (2018). How to play 20 questions with nature and lose: reflections on 100 years of brain-training research. Proc. Natl Acad. Sci. USA.

[3] Smid CR, Karbach J, Steinbeis N (2020). Toward a science of effective cognitive training. Curr. Dir. Psychol. Sci..

[4] Gathercole SE, Dunning DL, Holmes J, Norris D (2019). Working memory training involves learning new skills. J. Mem. Lang..

[5] Karni A, Sagi D (1993). The time course of learning a visual skill. Nature.

[6] Shiffrin RM, Schneider W (1977). Controlled and automatic human information processing: II. Perceptual learning, automatic attending and a general theory. Psychol. Rev..

[7] Ericsson KA, Chase WG, Faloon S (1980). Acquisition of a memory skill. Science.

[8] Klingberg T (2010). Training and plasticity of working memory. Trends Cogn. Sci..

[9] Schaie KW, Willis SL, Hertzog C, Schulenberg JE (1987). Effects of cognitive training on primary mental ability structure. Psychol. Aging.

[10] Schmiedek F, Lövdén M, Lindenberger U (2010). Hundred days of cognitive training enhance broad cognitive abilities in adulthood: findings from the COGITO study. Front. Aging Neurosci..

[11] von Bastian CC, Oberauer K (2013). Distinct transfer effects of training different facets of working memory capacity. J. Mem. Lang..

[12] Judd N, Klingberg T (2021). Training spatial cognition enhances mathematical learning in a randomized study of 17,000 children. Nat. Hum. Behav..

[13] Karni A (1998). The acquisition of skilled motor performance: fast and slow experience-driven changes in primary motor cortex. Proc. Natl Acad. Sci. USA.

[14] Ungerleider LG, Doyon J, Karni A (2002). Imaging brain plasticity during motor skill learning. Neurobiol. Learn. Mem..

[15] Pasupathy A, Miller EK (2005). Different time courses of learning-related activity in the prefrontal cortex and striatum. Nature.

[16] Tenison C, Anderson JR (2016). Modeling the distinct phases of skill acquisition. J. Exp. Psychol. Learn. Mem. Cogn..

[17] Gallistel CR, Fairhurst S, Balsam P (2004). The learning curve: implications of a quantitative analysis. Proc. Natl Acad. Sci. USA.

[18] Smith PL, Little DR (2018). Small is beautiful: in defense of the small-N design. Psychon. Bull. Rev..

[19] Molenaar PC (2004). A manifesto on psychology as idiographic science: bringing the person back into scientific psychology, this time forever. Measurement.

[20] Klingberg T (2005). Computerized training of working memory in children with ADHD-a randomized, controlled trial. J. Am. Acad. Child. Adolesc. Psychiatry.

[21] Alloway, T. P. Automated Working Memory Assessment. London: Pearson Assessment (2007).

[22] Gathercole SE, Durling E, Evans M, Jeffcock S, Stone S (2008). Working memory abilities and children’s performance in laboratory analogues of classroom activities. Appl. Cogn. Psychol..

[23] Cortes RA (2022). Transfer from spatial education to verbal reasoning and prediction of transfer from learning-related neural change. Sci. Adv..

[24] Johnson-Laird PN (2010). Mental models and human reasoning. Proc. Natl Acad. Sci. USA.

[25] Wai J, Lubinski D, Benbow CP (2009). Spatial ability for STEM domains: Aligning over 50 years of cumulative psychological knowledge solidifies its importance. J. Educ. Psychol..

[26] Hawes Z, Ansari D (2020). What explains the relationship between spatial and mathematical skills? A review of evidence from brain and behavior. Psychon. Bull. Rev..

[27] Mix KS (2016). Separate but correlated: the latent structure of space and mathematics across development. J. Exp. Psychol..

[28] Newcombe, N. Harnessing Spatial Thinking to Support STEM Learning Working paper 161 (OECD iLibrary, 2017). https://www.oecd-ilibrary.org/education/harnessing-spatial-thinking-to-support-stem-learning_7d5dcae6-en.

[29] Berger, E. M., Fehr, E., Hermes, H., Schunk, D. & Winkel, K. The Impact of Working Memory Training on Children’s Cognitive and Noncognitive Skills Discussion Paper No. 09/2020 (NHH Department of Economics, 2020). 10.2139/ssrn.3622985.

[30] Van De Schoot R, Schmidt P, De Beuckelaer A, Lek K, Zondervan-Zwijnenburg M (2015). Measurement invariance. Front. Psychol..

[31] McArdle JJ, Epstein D (1987). Latent growth curves within developmental structural equation models. Child. Dev..

[32] Roweis S, Ghahramani Z (1999). A unifying review of linear Gaussian models. Neural Comput..

[33] Holmes J, Gathercole SE, Dunning DL (2009). Adaptive training leads to sustained enhancement of poor working memory in children. Dev. Sci..

[34] Holmes J (2010). Working memory deficits can be overcome: impacts of training and medication on working memory in children with ADHD. Appl. Cogn. Psychol..

[35] Bergman-Nutley S, Klingberg T (2014). Effect of working memory training on working memory, arithmetic and following instructions. Psychol. Res..

[36] Roberts G (2016). Academic outcomes 2 years after working memory training for children with low working memory: a randomized clinical trial. JAMA Pediatr..

[37] Olesen PJ, Westerberg H, Klingberg T (2004). Increased prefrontal and parietal activity after training of working memory. Nat. Neurosci..

[38] Dahlin E, Neely AS, Larsson A, Backman L, Nyberg L (2008). Transfer of learning after updating training mediated by the striatum. Science.

[39] Kühn S (2013). The dynamics of change in striatal activity following updating training. Hum. Brain. Mapp..

[40] Constantinidis C, Klingberg T (2016). The neuroscience of working memory capacity and training. Nat. Rev. Neurosci..

[41] Erickson KI (2010). Striatal volume predicts level of video game skill acquisition. Cereb. Cortex..

[42] Ullman H, Almeida R, Klingberg T (2014). Structural maturation and brain activity predict future working memory capacity during childhood development. J. Neurosci..

[43] Todd JJ, Marois R (2005). Posterior parietal cortex activity predicts individual differences in visual short-term memory capacity. Cogn. Affect. Behav. Neurosci..

[44] Palva JM, Monto S, Kulashekhar S, Palva S (2010). Neuronal synchrony reveals working memory networks and predicts individual memory capacity Proc. Proc. Natl Acad. Sci. USA.

[45] Buonomano DV, Merzenich MM (1998). Cortical plasticity: from synapses to maps. Annu. Rev. Neurosci..

[46] Qi XL, Meyer T, Stanford TR, Constantinidis C (2011). Changes in prefrontal neuronal activity after learning to perform a spatial working memory task. Cereb. Cortex..

[47] Finc K (2020). Dynamic reconfiguration of functional brain networks during working memory training. Nat. Commun..

[48] Cubillo A (2022). Intra‐individual variability in task performance after cognitive training is associated with long‐term outcomes in children. Dev. Sci..

[49] Hertzog C, Lindenberger U, Ghisletta P, von Oertzen T (2006). On the power of multivariate latent growth curve models to detect correlated change. Psychol. Methods.

[50] Nolte S, Elsworth GR, Sinclair AJ, Osborne RH (2009). Tests of measurement invariance failed to support the application of the “then-test”. J. Clin. Epidemiol..

[51] Rosseel Y (2012). lavaan: an R package for structural equation modeling. J. Stat. Softw..

[52] Hu LT, Bentler PM (1999). Cutoff criteria for fit indexes in covariance structure analysis: conventional criteria versus new alternatives. Struct. Equ. Modeling..

[53] Chen FF (2007). Sensitivity of goodness of fit indexes to lack of measurement invariance. Struct. Equ. Modeling..

