# Supplementary material for: A dual-process model for cognitive training
Source: NPJ Sci Learn. 2023 May 6;8:12. doi: 10.1038/s41539-023-00161-2 (PMC10164188; doi:10.1038/s41539-023-00161-2)
Supplement: Supplementary file 1 — Reporting Summary [file 41539_2023_161_MOESM1_ESM.pdf]

nature portfolio

Corresponding author(s): Julia Ericson, Torkel Klingberg

Last updated by author(s): Feb 17, 2023

Reporting Summary

Nature Portfolio wishes to improve the reproducibility of the work that we publish. This form provides structure for consistency and transparency in reporting. For further information on Nature Portfolio policies, see our Editorial Policies and the Editorial Policy Checklist.

Statistics

For all statistical analyses, confirm that the following items are present in the figure legend, table legend, main text, or Methods section.

n/a	Confirmed	
☐	☒	The exact sample size (*n*) for each experimental group/condition, given as a discrete number and unit of measurement
☐	☒	A statement on whether measurements were taken from distinct samples or whether the same sample was measured repeatedly
☐	☒	The statistical test(s) used AND whether they are one- or two-sided<br>*Only common tests should be described solely by name; describe more complex techniques in the Methods section.*
☐	☒	A description of all covariates tested
☐	☒	A description of any assumptions or corrections, such as tests of normality and adjustment for multiple comparisons
☐	☒	A full description of the statistical parameters including central tendency (e.g. means) or other basic estimates (e.g. regression coefficient) AND variation (e.g. standard deviation) or associated estimates of uncertainty (e.g. confidence intervals)
☐	☒	For null hypothesis testing, the test statistic (e.g. *F*, *t*, *r*) with confidence intervals, effect sizes, degrees of freedom and *P* value noted<br>*Give P values as exact values whenever suitable.*
☒	☐	For Bayesian analysis, information on the choice of priors and Markov chain Monte Carlo settings
☐	☒	For hierarchical and complex designs, identification of the appropriate level for tests and full reporting of outcomes
☐	☒	Estimates of effect sizes (e.g. Cohen's *d*, Pearson's *r*), indicating how they were calculated

Our web collection on statistics for biologists contains articles on many of the points above.

Software and code

Policy information about availability of computer code

Data collection	The data were collected from the software Cogmed (www.cogmed.com), which is a cognitive training software designed for children.
Data analysis	The data were analyzed using R and python.

For manuscripts utilizing custom algorithms or software that are central to the research but not yet described in published literature, software must be made available to editors and reviewers. We strongly encourage code deposition in a community repository (e.g. GitHub). See the Nature Portfolio guidelines for submitting code & software for further information.

Data

Policy information about availability of data

All manuscripts must include a data availability statement. This statement should provide the following information, where applicable:

- Accession codes, unique identifiers, or web links for publicly available datasets
- A description of any restrictions on data availability
- For clinical datasets or third party data, please ensure that the statement adheres to our policy

Data availability: The data is available upon reasonable request to corresponding authors. Code availability: After publication, the code can be accessed at https://github.com/juliaericson/Working-Memory-Training-Learning-Curves.

Human research participants

Policy information about studies involving human research participants and Sex and Gender in Research.

Reporting on sex and gender	Sex and gender was not recorded.
Population characteristics	The only known information about the participant was age. The data set included 577 9-year-olds, 631 10-year-olds and 459 11-year-olds
Recruitment	Participants were not recruited for the study specifically. All children who had practiced the 20 min long Cogmed training program between 2016-2019 and were between 9-11 years old were included.
Ethics oversight	Swedish Ethics Review Agency.

Note that full information on the approval of the study protocol must also be provided in the manuscript.

Field-specific reporting

Please select the one below that is the best fit for your research. If you are not sure, read the appropriate sections before making your selection.

☐ Life sciences ☒ Behavioural & social sciences ☐ Ecological, evolutionary & environmental sciences

For a reference copy of the document with all sections, see nature.com/documents/nr-reporting-summary-flat.pdf

Behavioural & social sciences study design

All studies must disclose on these points even when the disclosure is negative.

Study description	Quantitative data was used and the comparison was longitudinal.
Research sample	The dataset included Cogmed users in the age group 9-11. No other information about the participants was available.
Sampling strategy	The data had already been collected prior to the study, and all available data was used in the study in order to have the maximum effect size.
Data collection	The data were extracted from Cogmed's database. The data was not collected with a researcher present.
Timing	Data were collected between 2016 - 2019.
Data exclusions	366 children who had missed two or more test sessions out of five were excluded. Furthermore, one subject was excluded from the second analysis (HMM) as one of the estimated values for this participant was 10 times larger than the standard deviation of the population.
Non-participation	Of the 1,667 children that started, 366 children did not complete at least four of the five test sessions.
Randomization	Participants were not divided up into groups.

Reporting for specific materials, systems and methods

We require information from authors about some types of materials, experimental systems and methods used in many studies. Here, indicate whether each material, system or method listed is relevant to your study. If you are not sure if a list item applies to your research, read the appropriate section before selecting a response.

Materials & experimental systems

n/a	Involved in the study
☒	☐ Antibodies
☒	☐ Eukaryotic cell lines
☒	☐ Palaeontology and archaeology
☒	☐ Animals and other organisms
☒	☐ Clinical data
☒	☐ Dual use research of concern

Methods

n/a	Involved in the study
☒	☐ ChIP-seq
☒	☐ Flow cytometry
☒	☐ MRI-based neuroimaging
